# Medical News

**Published:** 1853-09

**Authors:** 


### MEDICAL NEWS.

[The following beautiful tribute to Dr. CHAPMAN was addressed to him many years ago, by his early friend, Dr. J. K. MITCHELL, who afterwards accepted and still worthily fills in the Jefferson Medical College, the same chair which was illustrated for so long a period by Dr. Chapman in the University of Pennsylvania. Its republication, at the present time, will, we are satisfied, be interesting to our readers.]

DEDICATED TO N. CHAPMAN, M. D.

Dear Doctor, though I hae the will,
I fear I want poetic skill
To do ye muckle credit;
But yet I'll imp my youthfu' wing,
And o' my guid preceptor sing,
Though ye y'ersel may dread it.

I've aften wished for Burns's pen,
And thochts frae Ramsay's fairy glen,
To do ye fitting honor,
But tak the will and no the deed,
My muse, the jade, awa will speed,
Sae I maun e'en get on her.

Ah ! weel I mind when first I saw
Ye laying down the morbid law
O' nature to the student ;
To dry detail and dusty lore,
Brocht frae y'er inexhausted store,
A new enchantment you lent.

Frae worthies o' the aulden time,
To those wha yet were i' their prime,
Ye drew y'er rich resources ;
And last, not least, frae y'er ain sel,
Baith thochts and words o' magic spell
Adorn'd y'er ripe discourses.

Wi' easy grace and potent sense,
Clear order, a' without pretence,
And learning without show, sir,
Ye charm'd the eye, and pleas'd the ear,
And made y'er thochts sae richly clear,
The darkest truth did glow, sir.

But faith, I scarce believ'd my eyes ;
Ye took me, sir, wi' sair surprise,
When mang y'er friends I saw ye
Let loose the wit by science chain'd—
Humor that nae ane ever pain'd—
Oh ! thus I'd like to draw ye !

They little ken ye wha hae known
Y'er science and y'er skill alone,
Though they are mair than ample ;
The racy pun, rich repartee,
The gushing joke frae malice free,
Wad na complete the sample.—

But better far, a heart that ne'er
Did o'er a human ill forbear
To heave a feeling sigh,
That readily forgave a foe,
And never dealt a jealous blow,
In keenest rivalry.

Mair I might say, but this I fear
E'en frae a friend ye'll hardly bear,
Sae I'll nae mair offend ye;
Though if ae man beside y'ersel
Says that the truth I dinna tell,
That man has never kenn'd ye.

J. K. Mitchell.

Comparative Healthiness of England, France, Prussia, &c., —The annual rate of mortality in England may be best understood by saying that there are 46 persons living to 1 dead. That is, the mortality as 1 in 46. In 1841, the annual mortality per cent. was 2207, or 1 in 45. In France there were 42 living to 1 death; in Prussia, 38; in Austria, 33; and in Russia, 1 person in 28 died annually. In the latter country, the mortality per cent. is 3590, or in other terms, out of every 100,000 Russians living, 3590 died in one year; while out of ever 100,000 British living, only 2507 died in the same period. In several Italian cities the annual mortality of the inhabitants is from 3 to 4 per cent.. In the city of Naples, which has been shown to be one of the unhealthiest in Europe, it appears that 4046 persons died out of every 100,000 of its inhabitants. From these facts, it is evident that there is a lower rate of mortality among the inhabitants of this country than in either of those enumerated, and that in some instances the mortality falls little short of one-half the same as existing in less favored regions.—*Lancet.*

The Jenner Testimonial.—It is fully determined to execute with every possible despatch the well-merited testimonial to the memory of a man whose discovery of vaccination has immortalized him. With that object subcriptions are being raised throughout every kingdom. The United States raised for that purpose 1600 dollars,—a larger sum than any other country subscribed.—*Ibid.*

Asiatic Cholera in Copenhagen.—A London daily paper states, "We have received information that this dreaded disease has re-appeared in an epidemic form in Copenhagen, where cases of it have existed, proving rapidly fatal ever since the middle of June. From that period the disease has been slowly but steadily increasing; and the official return for the 8th of the present month states that on that day there were 51 new cases and 18 deaths. From the first appearance of the disease, there have been registered in all 270 attacks and 142 deaths; so that this formidable pestilence, which for several months has been lingering in St. Petersburgh, has now approached to within less than 500 miles of the coast of Norfolk. On Tuesday last there were 53 new new cases of cholera, and 36 deaths. Since the 12th of June, 427 persons have been attacked, and 234 have died.—*Ibid.*

Frightful Mortality in Calcutta.—Owing to the great heat and the absence of rain, eleven hundred persons were carried off, at Calcutta, in the course of two days.—*Lancet,* from *Overland Athenæum.*

Royal Acknowledgment of Medical Services.—On the 8th August last, her Majesty was graciously pleased to confer the honor of Knighthood upon Dr. John Forbes, and Dr. James L. Bardsley, of Manchester.—*Lancet.*

The Medical Chronicle, or Montreal Monthly Journal, edited by William Wright, M. D., and D. C. Mac Callum, M. D., made its debut in June last. It promises to be an interesting and spirited journal.

The first number of the Medical Reporter, a quarterly Journal, published under the direction of the Chester and Delaware County Medical Societies of Pennsylvania, was issued in July. The editorial committee consists of Drs. Wilmer Worthington, Isaac Thomas, and Jacob Price, of Chester county, and Drs. George Martin and John T. Huddleson, of Delaware county.

The Astley Cooper prize of £300 for the best Essay on the "Structure and Functions of the Human Spleen," has been awarded by the physicians and surgeons of Guy's Hospital to Henry Gray, Esq., F. R. S., Wilton-street, Grosvenor-square.

The Cholera.—The Dutch Government have received the official notification from the Dutch Minister Plenipotentiary at Stockholm, that the Swedish Government have declared that the cholera prevails in Abo, Elsinore, St. Petersburg, Cronstadt, Narrva, Reval, Riga, and Copenhagen, and that the following places and territories are "suspected to be infected:"—All the Finnian harbors from Christianstadt inclusive to the Russian frontiers; all the Russian ports of the Gulf of Finland and the Baltic, and the ports of Zealand. Copenhagen, July 31.—The cholera seems to have diminished here somewhat in intensity, as there are only 237 new cases, and 115 deaths. Up to the present time the 45th part of the population has been carried off by the malady. Our accounts from Copenhagen, up to the 4th instant, show the cholera to be somewhat on the decrease, the new cases seldom exceeding 150 per day. The whole number at present announced amounts to 5996, with 3219 deaths. It is to be observed, however, that now that the disease has attacked the middle-classes, who, for the most part, were treated at their own houses, the number of cases published would always be considerably under the truth.

The Cholera.—The last news from Persia announces, that the cholera is making great ravages in Astraband, Mazraderam, and the desert of the Turcomans. In one of these provinces, the number of deaths amounted to 150 a day. The Shah and his Court had retired to Imama. To complete the distress, the towns of Shiraz and Surham had been nearly destroyed by earthquakes, the Zaindowod was dried up, so that, from its muddy bed, innumerable bands of grasshoppers proceeded, destroying everything in their progress.

Death of Dr. Pereira.—We are permitted to extract the following reference to the death of this distinguished physician from a private letter to Dr. Duck of this city. The writer says: "Poor Pereira, his loss is felt by all; he was buried at the Kensalgreen cemetery, and was followed by the whole school of the London Hospital and also by between thirty and forty practitioners, many of them, including myself, coming a distance of between six and seven miles. Dr. Billing kindly took me in his carriage coming home, when I had the advantage of hearing his opinion as to the cause of death. Dr. B. thinks that when Dr. Pereira slipped on the back stair case of the College of Surgeons, in addition to the rupture of the tendon of the left quadriceps extensor femoris, he also ruptured the two inner coats of one of the sinuses, at the origin of the aorta, and that on exerting himself to relieve his attendants while lifting him into bed, a fortnight afterwards, he ruptured the remaining coat and died from internal hemorrhage. His friends objected to a post-mortem examination, and it was not urged. A committee of four or five physicians are completing the second part of the 2d volume of the learned Doctor's Materia Medica, which Dr. Billing says is a perfect encyclopedia. The greater part was already finished and in the press."—*N. W. Journal, August.*

Death of William Beaumont, M. D.—He died at his residence in St. Louis, on the 25th of April last, in the 68th year of his age. Dr. Beaumont was a native of Lebanon, Connecticut, where he was born in 1785. In 1812, after studying medicine at St. Albans, Vermont, for two years, he joined the Sixth Infantry, with the appointment of Assistant-Surgeon. For more than twenty years he was a member of the medical staff of the regular army, being stationed at various points on the northern frontier, and through the war of 1812, with distinction—being present, among other occasions of interest, at the capture of Fort George, in May, 1813. In 1830, he was stationed at Jefferson Barracks, and afterwards in the Arsenal at St. Louis, taking up his residence in this city about 1834. Two or three years later he resigned from the army, and subsequently has resided constantly in St. Louis, enjoying an extensive practice and high professional reputation up to the period of his late illness.

Dr. Beaumont is widely and most honorably known in the literature of our profession by his "Physiology of Digestion and Experiments on the Gastric Juice"—based on and containing an account of experiments conducted by himself upon a Canadian (Alexis St. Martin,) whom he attended at Michilimackinac, 1825. The work has been reprinted in Great Britain, France, and Germany, with the highest commendations from the profession, and has become an acknowledged authority in matters where speculation had hitherto taken the place of observation.

Dr. Beaumont had lived in St. Louis for many years, engaged in arduous professional duties, and wherever he was known, it was as the kind-hearted, generous gentleman. In his social relations, he was most happy, diffusing at all times cheerfulness and contentment to those around him.—*N. Y. Journ. Med.*

Death of G. W. Richards, M. D.—Dr. Richards died at Dubuque, Ia., on the 22d of April, aged 53 years. He was for a short time connected with the Rush Medical College in Chicago, as one of its Professors, and afterwards became Professor of the Theory and Practice of Medicine, in the Medical College at La Porte, Ind. At a later period, he assisted in organizing a Medical School at Rock Island, and but two weeks before his death, was elected President of the Northwestern Medical Society, organized at Dubuque.—*Ibid.*

Death of Charles Caldwell, M. D.—From the Louisville (Ky.) Courier, we copy the following synopsis of the principal events in the life of Dr. Caldwell, whose decease took place in July last, together with some just comments on the literary and scientific character of that remarkable man.—"He was about ninety years of age, and probably the oldest physician in the United States, and enjoyed the greatest celebrity as a medical writer and teacher. He did more than any one to enlighten the public and the profession on the origin of yellow fever, and clearly illustrated the absurdity of quarantines. Some years before the Asiatic cholera invaded Europe as an epidemic, Prof. Caldwell predicted that in one respect cholera would prove a blessing to mankind, by teaching the worthlessness of quarantine regulations, and the vital necessity of attention to all the laws of sanitary science; and this prediction, as full and clear as the history of the epidemic can be made now, has been verified in every particular. This prophetic prediction of the venerable teacher was recently made the conclusion of an invaluable report on cholera, published by the British Parliament.

"At the commencement of his medical career, Prof. Caldwell settled in Philadelphia, and won great distinction. Among the writers and investigators of that period, Dr. Caldwell was the greatest. He towered above his contemporaries, as a tall monument springs from the plain.

"In addition to Dr. Caldwell's luminous and voluminous labors upon all the important questions of medical science, all subjects of public interest felt the benefit of his intellect. His papers on Quarantines, Malaria, and Temperaments, are among the best in the English language on those topics. His treatises on Physical Education, on the Unity of the Human Race, and on Phrenology, have rarely been equalled. Every thing he touched he adorned. We doubt whether the English language contains a biographical sketch equal to Dr. Caldwell's tribute to Fisher Ames, published in the American edition of Rees's Encyclopedia. A recent edition of his work on the Unity of the Human Race, displays a remarkable instance of intellectual vigor in one who had passed that period at which mental power usually begins to falter. In that work Dr. Caldwell reviewed a recent work on the Races by Dr. Knox, of England, and the criticism is one of the ablest and most conclusive we know of. Quite recently, Dr. Caldwell published a paper in the Western Journal of Medicine and Surgery on Liebig's theory of Animal Heat; and the distinguished Professor of Giessen has not received such a blow from any other quarter. But time and space would fail us if we were to attempt an enumeration in this paper of the works of Professor Caldwell.

"The great reputation of Dr. Caldwell as a medical scholar, teacher and writer, induced the friends of western enterprise in medical teachings, to invite him, about 1818, to a chair in the Transylvania School of Medicine. He accepted the trust, and entered upon the discharge of its duties with a zeal, intelligence and power that were determined to know no such thing as failure. He was the bright particular star of Transylvania, and during his connection with the institutien it prospered. The labors of himself and of his colleagues, who caught inspiration from his example, made the Transylvania School of Medicine equal to any in the Union, and he had much to do with its proud pre-eminence.

"When he discovered that the spirit of the age demanded means for clinical instruction, and a larger field for medical observation than a village could furnish, he promptly entered into arrangements for transferring the Transylvania School to this city. Upon the failure of that attempt, he entered zealously into the project for establishing a school of medicine in Louisville, and by his labors, talents and eloquence, the project was forwarded. And to the same great powers the school was mainly indebted for its remarkable success.

"Dr. Caldwell was one of the most temperate men we have ever known. His science enabled him to keep a true sentinelship over his appetites, and the result was an exceedingly long life, far beyond that allotted to man by the royal Psalmist, with an almost entire exemption from sickness. Even in the closing scenes of life, disease did not invade his frame. He was nearly free from physical suffering; all the functions of his system were as well performed on his death-bed as during his highest health, and his mind was clear to the last. His life and death are impressive commentaries upon the truth of physiological doctrines which he taught for half a century, and by which he regulated his life and ordered its last scenes."

Medical Institutions of Berlin. By W. H. Hingston, M. D., L.R.C.S. Ed., Honorary and Corresponding Member of the German Medical Society of Paris.—It is often a question as to which of the medical schools of Germany holds the highest rank. The question is one of importance, as it has frequently to decide or regulate the time to be spent in each by those seeking professional education; yet is it one which cannot be answered generally or definitely. Thus, Prague and Vienna have long held pre-eminence in midwifery. More recently medical pathology is a branch which has been cultivated with great success in the latter. Physiology has made rapid strides in Berlin, Wurtzburg, and Bonn; Giessen has advanced chemical science, &c., &c.; but when speaking of surgery alone, the question is divested of most of its difficulty.

In this department of science, Berlin has for some time stood pre-eminent, and still claims the foremost rank. During the residence here of Graeffe, surgeons and students from all parts of the continent, and many from Britain and America, were attracted to the Prussian capital. His world-renowned successor, Dieffenbach, added new lustre to the chair (already famous) until death removed him also from the scene of his usefulness and success. It was here tenotomy was first extensively and and successfully practised.* It was here also rhino-plasty was first given to the world. Graeffe's success in practice was very great. Dieffenbach's memory is still fondly cherished by the great mass of the people. Indeed it would be difficult to conceive greater affection for one nearly four years dead. The rich speak of him in terms of fondness, respect, and praise; the poor mention his name with reverence. Like John Hunter, his death was sudden and unexpected, and took place in the theatre of his greatest usefulness. One day when seated on a sofa, observing a student making an examination of a patient, he quietly expired. He was attended to his final resting-place by thousands. His requiem was the prayers and lamentations of those to whose comfort he had ministered. Dieffenbach was succeeded by Langenbeck; but I shall mention the present surgeons in connexion with the various institutions to which they belong.

*The University.*—A very fine building, built by Fred. Wm. III. opened in 1810, when, by order of the Prussian government, or rather the king, the University was removed from Frankfort-on-the-Oder to its present situation—Berlin. At first the number of students who were attracted to the Prussian capital was not very great, for although professors of acknowledged ability were here to be met with, yet the advantages afforded to military over civil students proved extremely distasteful to the latter, and prevented many from attending its sessions.† By a wiser policy of government, by gradually yielding to the wishes of the people, and by granting to civilians all the privileges they accorded to others, the University began to flourish. At the present moment it

* Tenotomy (by subcutaneons section) was first practised by Stromeyer of Kiel; but to Dieffenbach is due the credit of having brought it prominently before the British public.

† These remarks apply only to students of medicine. On those studying arts, law, or divinity, were placed no restrictions, for such are not usually educated for the army.

numbers 2200 matriculated students; of these about two-ninths are medical.

There is no school in Germany where so many students are collected together. Prague, the oldest, has 1272; Munich, 1957; Bonn, 866; Wurtzburg, 722. It would occupy too much space to enumerate all the German universities, amounting to twenty-four, and their statistics. I have mentioned these merely to show the flourishing condition of the one about which I am writing. Labor is there divided among the various professors in their several departments. In theology there are five ordinary, and four extraordinary professors, besides two *privatim docentes;* In law, nine ordinary, four extraordinary, and three *privatim docentes.* In the department of medicine there are eleven ordinary, six extraordinary, and nineteen *privatim docentes.* In philosophy twenty-seven ordinary, twenty-nine extraordinary, and thirty-one *privatim docentes.* In modern languages five.

The largest building for the relief of the sick is the Charité. It does not belong to the University. It was built by Fred. Wm. I. entirely for the education of surgeons for the Prussian army. It continued as such till 1810, when the University was brought from Frankfort-on-the-Oder to Berlin. Previous to that time there were professors of the Military Academy, who gave lectures to young men studying for the army. In 1810 the professors of the University were allowed wards for their clinique, and students then matriculated. In 1848 government made still greater concessions, by permitting civilians to become assistants to the professors in one medical clinique. The Charité, governed by two directors, one medical and one administrative, is divided into old and new. In all, it contains 900 beds, but could accommodate nearly double that number in urgent circumstances. The new contains all the syphilitic patients, insane persons, and prisoners, and also those sent from the prisons. The old contains medical, surgical, and obstetrical cases, and children.

From the medical wards, four are selected for the clinique, two male and two female, one for each professor. From the surgical are taken four wards for clinique, two of which are for ophthalmic surgery. These are attended by the same professor. Drs. Schoenbein and Wolff are the professors of medicine. The former, although pretty well advanced in years, is still active. From œdema glottidis, or some other affection of the throat, he is with difficulty understood. Strangers to the language or those who do not understand it thoroughly, will derive no advantage from attending his instructions, as a large portion even of his own countrymen find great difficulty in following him. His therapeutics, however, are excellent. He has been the king's physician for years, and is highly esteemed by the profession. A work appeared many years ago, under the title of "Therapeia von Schoenbein," but he denied the authorship· I believe some students had taken notes of his lectures, and published them in a continuous form. His assistant, Dr. Traube, devotes several hours a day to giving instructions on auscultation and percussion. He has written many excellent papers; one in particular, on "Critical Days," is highly valuable. Inenken is the professor of surgery. High praise cannot be bestowed either on his operations or after-treatment; tedious in the former, meddlesome in the latter. He has been rather unsuccessful in the administration of chloroform. *On dit* that he has lost seven patients in this way. As it is an *on dit*, it might be well to take it *cum grano salis.* I saw a patient in January last, from whom he was removing the orbit, expire on the table. Inenken attributed the death to the shock of the operation, as he had done on previous occasions. This theory he has endeavored to maintain publicly, in opposition to Langenbeck, but much against the notions of the profession generally. As an oculist, Inenken holds, and deservedly too, a very high rank. He operates on the eye with care, dexterity, and precision. His diagnosis is almost infallible, his after-treatment good. He still holds to the old operation for cataract—the low incision. In this respect I believe he stands alone.

The obstetrical department, by a very unwise and prejudicial arrangement, is open to students only in the summer season, and to midwives in the winter. There are but thirty beds, which are seldom fully occupied. About 400 births take place in the year. Berlin makes no pretensions to midwifery; her claims are more easily substantiated in consequence. Young military surgeons are the resident accoucheurs in this branch.

Dr. Simon has the charge of the syphilitic patients. He is also lecturer on syphilis and diseases of the skin in the University. His works on those subjects bear evidence of considerable research. The females in the syphilitic wards are examined three times a week, the men twice. Those who have witnessed the examination of females in the hospitals of Britain, might be somewhat shocked at the manner in which patients are required to mount the steps, and uncover their person from the abdomen downwards. This is both extremely indecent and demoralising, the more so as it is unnecessary.

Apart from the Charité, and in connexion with the University, are three cliniques for medicine, surgery, and midwifery. They are all supported by government, and consequently admit patients from all parts of

Prussia. Dr. Busch is the professor of midwifery. He is not much seen by the students, except at lecture. He has gained his laurels, and now seems to enjoy his *otium cum dignitate.* Besides these, there are polycliniques for each of these branches. Those patients who are not desirous of entering hospital, can be attended at their own houses by the students, and when necessary, by the assistants. In the hospital of the University about 300 births take place in the year; in the polyclinique between 800 and 900.

It was in the polyclinique in Ziegel Strasse that Graeffe gave instruction. It was here, also, where Dieffenbach held forth; and what is still more to the purpose, it is here that Langenbeck may be seen daily, with a large number of cases for consideration. In no clinique I have yet visited, have I seen so many operations as are here to be witnessed. The hospital is not a large one, but only such cases as demand operation are admitted. Almost every day, persons in the city and neighborhood are brought to be operated upon, and carried back again to their respective domiciles. It has been stated that there are more operations performed here than in any hospital on the Continent. Certainly Paris has nothing to equal it. The reputation of the operator draws to the capital persons from all parts of Prussia and surrounding duchies. As a lecturer and teacher, Langenbeck is considered superior to his predecessor. As I have not seen any similar treatment in British hospitals, I will occupy a few lines in speaking of anchylosis generally.

In the words of Cooper, anchylosis denotes an intimate union or growing together of two bones, which were naturally connected by a moveable kind of joint. In the true, the bones grow together so completely, that not the smallest degree of motion can take place, and the case is positively incurable. In false anchylosis the bones have not completely grown together, and their motion is only diminished, not destroyed. Anchylosis is generally looked upon as a favorable and in many cases a desirable termination of inflammation or ulceration. It is thought to be as near an approach to health as it is possible, under many circumstances, to attain; or in the words of Miller, it is a compromise between health and disease. Cooper remarks, "No attempt should ever be made to *cure*, though every possible attempt should often be made to *prevent* a true anchylosis." The exertion to prevent, he further adds, is not always proper, for "many diseases of joints may be said to terminate when anchylosis occurs."

With such authority before us, and the authority of many more I might quote, it would require no small amount of courage to interfere with what has been designated "a favorable termination," "a desirable termination;" and a surgeon who would undo this wise provision of Nature, might be pronounced reckless and bold. I confess that such would have been my own opinion had I read and not seen the treatment—the successful treatment, of *true*, and in many cases long standing, anchylosis, by breaking up the callus. This mode of practice is not novel. It was tried many years ago in France, but the result of these experiments was such as to discourage others from repeating them. Violent inflammation of the joint, and the most disagreeable consequences, generally followed; and the patient was left in a worse condition than previous to those experiments. But the French erred greatly. Instead of breaking up the callus gradually and cautiously, they restored the perfect flexion and extension of the limb immediately, and then kept up the motion of the joint, in order to free the condyles of the abnormal adhesions. As already remarked, the consequences were frequently very serious. The majority of surgeons would not follow the example, and writers on surgery only mentioned this mode of treatment in order to caution the profession against adopting it.

The practice so successful under Langenbeck, and which has given rise to those remarks, consist in breaking up the callus certainly, but in a manner very different from that employed by Louvrier. The patient is placed upon his back, and chloroform administered until complete anæsthesia is induced; until, in fact, *the muscles cease to offer any resistance.* If the anchylosis be of the knee (the most favorable joint for the operation) an assistant, or assistants, fix the pelvis, and the surgeon commences gradual flexion, if the joint be in an extended state, or extension if in a flexed state. The force to be employed must be regulated by the surgeon himself. In cases of many years standing, the force required, as might be supposed, is considerable; sometimes the whole weight of the body is necessary, but it must always be applied with care. When the callus yields to the external force, the amount of flexion is preserved until the next trial, the limb being exercised, passively, during the interval. A great degree of flexion is not to be desired at once; and the more cautiously and patiently the limb is managed, the less danger of violent reaction, and the greater probability of success. Indeed, this constitutes the great difference between the treatment of Louvrier and that of Langenbeck, or between failure and success. When managed with due care, the inflammation set up is very trifling, indeed seldom sufficient to retard the cure. Frequently no reaction is perceptible. Every two or three days the patient is again subjected to the same treatment. Sometimes a couple of months are necessary to restore the function of the joint perfectly, but three or four weeks are frequently suffi cient. Although many years may have elapsed between the occurrence of the anchylosis and the attempt at restoration, yet in most cases the callus may be broken up, and the integrity of the joint restored. It is absolutely requisite, however, that the patient should exhibit no tendency to scrofula, and this the more especially if the anchylosis be the result of scrofulous inflammation or ulceration.

From the want of exercise of the joint, the limb is usually much smaller than the sound one. Sometimes almost total atrophy of the muscles is the consequence, but a short time suffices to restore them to their original volume. In concluding these remarks, I feel perfectly justified in supporting the claims this mode of treatment has to be admitted among the regular operations of surgery.* And as anchylosis, as a result of disease of, or injury to the joint, is unfortunately of frequent occurrence, any mode of alleviating the subjects of it should obtain a fair trial, especially when supported by experiments.

In the same building in Ziegal Strasse, Romberg holds his clinique. He is particularly distinguished for his treatment of nervous affections. His admirable work on "Diseases of the Nervous System," said by some to be somewhat unintelligible, has been translated into English, and is no doubt familiar to many. There are several private institutions for the education of young men that have no support from government, the professors of which are recognised by the University as extra-academical professors, or *privatim docentes.* I will only mention those of excellence. In the Orthopædic Institution of Dr. Buehring, almost every kind of deformity may be seen, and an infinite variety of apparatus. A few months' attendance will suffice to show the most sceptical the great advantage which frequently attends the judicious employment of mechanical means for removal of deformity. I have there observed, and only there, curvatures of the spine in young girls, deformities of the femur, &c., brought back to their original condition, while, at the same time, the young patients were daily improving in health, and they have finally left the hospital with rosy cheeks and robust health. The Doctor confines them to the horizontal posture, only three or four hours daily. Some of his little patients have been with him twelve, and some eighteen months. Buehring has strongly advocated the bringing together by force of the two superior maxillaries in congenital absence of the hard palate. He

*I have devoted more time to this matter than I originally intended; but as it is an important one, and the mode of treatment peculiar to Berlin, I thought it entitled to more than a passing remark.

was the first to practise and recommend this operation. Dr. B. is a nephew of the late Dieffenbach, and edited his work on "Operative Surgery," which appeared shortly after his death.

Dr. von Graeffe has a clinique for diseases of the eye. A great collection of cases may be seen at almost any hour, but especially from nine to eleven A. M. He enters very fully, when occasion presents itself, into the physiology of the eye, and the laws of optics. I may mention the treatment of ulcerations of the cornea, by the solution of atropine, which I have seen applied almost daily, with the greatest success. It is not necessary to allude to all the remedies recommended. Nitrate of silver in substance, weak and strong solutions, and sugar of lead in powder, weak and saturated solutions, seem to be the favorites with the profession. Jacob, in his "Essay on the Morbid Anatomy of the Human Eye," p. 367, says that caustic sometimes becomes permanently fixed, and constitutes an indelible dark speck. The same author, p. 369, makes an equally grave charge against the use of acetate of lead. He says that the obstinate and intractable nature of some ulcers, whose surface presents an opaque white color, is due to the employment of the acetate of lead. He seems to think that the acetate is decomposed, and that a white precipitate is deposited in the ulcer, to which it adheres tenaciously, and in the healing becomes permanently and indelibly imbedded in the structure of the cornea. To atropine in solution there can be no such objection. It cannot leave any dark speck or precipitate to be imbedded. Whether it exerts a specific action on the ulcer, or acts indirectly through the capillaries, I cannot say; but certain it is, that ulcers have been found to heal under this treatment much more rapidly than under any other local application. I merely mention the fact, and leave it to others to determine its action. Dr. Graeffe employs it also in conjunctivitis. Dr. G. is son of Dr. G. whose name I have already mentioned. The money left by his father enables him to support an hospital at his own expense. His acquaintance with French and English, and his desire to impart instruction, by speaking in either language when necessary, renders him a favorite, and his clinique is much frequented by persons speaking these languages.

The following are not intended for the education of students:—Bethanien, founded by Frederick William IV., and opened in 1848, for the education of Protestant Sisters of Charity as nurses (Diakonissen), who here serve a noviciate of one year before becoming "sisters." Those sisters are not composed of the lower classes, but frequently belong to the first families of Prussia.* Unlike the sisterhood of the Roman faith, they pledge themselves to a life of celibacy but for five years, at the end of which, if Providence has thrown in their way some likely fellow, they can doff the modest gray and white; if not, they may renew their vow for other five years, or for life. They are paid a trifling sum yearly, barely sufficient for clothing. They frequently serve in other hospitals, and in the city, as nurses. The proceeds of their services is paid into the treasury of the Bethanien, and they return to their Mütter Haus. Like most establishments, where sisters of charity are nurses, everything is very clean, and kept in the best order. The hospital contains but three hundred beds. I have seen three times that number confined in a smaller space. The roof is high, windows large, rooms light and cheerful, floors beautifully polished, passages heated with air, by pipes brought from the furnace. There are also pipes to convey away the foul air, and introduce fresh. Each ward contains from twelve to sixteen beds, having sometimes the length of a bed between them. There are four physicians and surgeons. The directors and apothecary are both females, elected from among the Diakonissen.

Elizabethan: for diseases of women, generally contains ninety beds—also attended by sisters of charity.

Kinder Hospital: for children of from 1 to 11 years of age.

Armen Haus Hospital: far vagrants, drunkards, incurably sick, incurably insane, and prostitutes, who are all kept separate. It contains one thousand beds, which are well occupied in winter, but in summer rather empty.

Before taking leave of the various hospitals, I feel bound to mention, in the warmest terms, the admirable appointments that distinguish them. Most of them are fitted up with every care to the comfort of the inmates, some of them with elegance, at least elegance as compared with what we usually meet in institutions of a like nature. The most casual observer cannot fail to notice the very great cleanliness and comfort that reigns throughout. Light, airy, and cheerful (one might easily forget he was in an hospital), they form an excellent contrast to the dark, dreary, dismal, overcrowded and ill-ventilated dungeons of the so-called head of affairs medical, Paris; for there, as in many parts of Britain, these are luxuries to which the eye is unfortunately not much accustomed.

When writing of medical affairs in Berlin, it will not be out of place

* The Countess of Stolberg, recently married from this Institution, had been a nurse for many years.

to mention some of the most striking features that distinguish the medical police of Berlin, and Prussia generally; for the same wise and salutary laws for sanitary purposes, are applicable to the whole Prussian kingdom. I select Berlin for the sake of convenience merely. It is divided into a number of wards or sections, each of which is attended by a medical man, who is paid a regular salary for attending to all those residing in his section, who may require his services. His prescriptions are sent to an apothecary, who charges the medicine to the town, and the poor are thus free of expense. All tradesmen, engineers, blacksmiths, carpenters, &c., are compelled to pay a small sum weekly when in service, for the help of their own sick. This originated with themselves, in a bond resembling Odd Fellowship, but has now become a law. The tax is regulated according to their wages, and is deducted from their weekly pay.

There exists another society, which is not fully under the surveillance of the police; and which, on account of its independent nature, stands great chance of being broken up. This is the Hygienic Association. It numbers upwards of 10,000. Members by paying two groschens (about $2\frac{1}{2}d.$) monthly, receive the services of a medical man during health and sickness.* It is necessary to announce the birth of every child to the police, and to state whether born healthy or diseased. After some months it must be vaccinated. For this purpose there are institutions in every town, and at convenient distances in the country. Every Prussian must serve three years in the army, and when enlisted he is re-vaccinated. No person is employed in any service, civil or military, without certificate of vaccination. There are a few houses licensed by the city, which are visited twice a week. If the police have reason to suspect that fair damsels receive the nocturnal visits of persons *illegitimately*, they are waited upon and examined, and compelled to go to the Safety Committee. The detection of these is rendered easy by the fact that all houses licensed are situated in a certain quarter of the city, the Koenigs Mauer, and the inmates are not allowed to extend their haunts beyond it. I may here remark that the medical police are in connexion with, but over the town police.

Besides the physicians already mentioned, there are eight for the city, whose duty it is to visit manufactories, workhouses, gaols, boarding-schools, &c., to see that the inmates, have comfortable apartments and

* This association has since been demolished by the police. It was thought that the principles of hygiene they wished to introduce extended even to government; that it was, in fact, a secret political society.

sleeping rooms, sufficient clothing, and food of a proper quality; that they have their regular hours of recreation, and that they are not tasked either in work or study beyond their strength.

Physicians must announce to the police all the cases of cholera, small-pox, epidemic dysentery, or supposed infectious disease, that may occur in their practice, scarlatina and measles excepted. They must also give to the parents or friends of any one dead in their practice, the date of death, and the name of the disease of which he died. Every week a list is published of all the deaths, with the diseases subjoined. The names of the deceased are published in connexion with the church to which they belong. I shall now notice very briefly the curriculum of the study of medicine in Prussia.

Before being allowed to matriculate, students are compelled to undergo an examination in classics, French mathematics, history, and German literature. They have to write a thesis in Latin and German, on any subject given them. The principles of philosophy, logic, and metaphysics, are also entered into. This past, they are admitted to the University. Their study must extend over a period of four years, or eight *semestres*, four of which must be in a Prussian university. At the expiration of two years, they are examined in physics, chemistry, botany, zoology, mineralogy, and some branches of philosophy. This examination is necessary before proceeding with further studies. These examiners are not connected with the medical faculty. At the end of the four years, they are examined by the medical faculty. The examination commences with a thesis prescribed by the dean of faculty. When written and approved of, the dean, who is annually elected from among the professors, examines the candidate on medical subjects generally. If successful, each professor examines him in his particular department. This is called *examen rigorosum*, but in comparison with what is to follow it is not rigorous. He has then to write a thesis in Latin, and print, at his own expense, a sufficient number of copies to enable him to send one to every university in Germany, and three to each professor or teacher attached to the University to which he belongs. This thesis is impugned, and must be defended against three adversaries. The debate, which is not usually very animated, is conducted in Latin, and is public. This over, the title of M. D., is conferred.

But in order to be enabled to practise, a *staats examen* or *cursus* must be undergone. It commences about six or twelve months after, but may be deferred as long as the candidate wishes, and usually lasts five or six months. In this cursus the following branches are gone over:

anatomy, surgery, medicine, and midwifery. In clinical medicine and surgery, the candidate from time to time receives patients, and in presence of professors and students make the diagnosis, offers a prognosis, and prescribes the treatment. He is then locked up, and has to write a complete history of the disease. In midwifery the candidate makes an examination of several women, and states the stage of pregnancy, &c. He has also to make deliveries in presence of the professors. On the dead subject, he has to go through all the operations—amputations, ligatures, &c., besides the more elementary parts of surgery, bandaging, &c. In anatomy, the candidate is required to make a preparation of any part given him, and of any system.

The *staats examen* from beginning to end is conducted by lottery. The candidate draws from a number of others a paper, on which is written what is required of him. He draws in each branch separately.—*Glasgow Med. Jour.*

COMMENTS ON THE LATE PROCEEDINGS OF THE AMERICAN MEDICAL ASSOCIATION.—The following spicy notice of a portion of the Proceedings of the American Medical Association, is from the Virginia Medical and Surgical Journal. We copy it, to show the feeling in an independent and respectable quarter, as regards the "*masterly inactivity*" policy of the Association on the subject of medical reform:

"We come now to the measures of medical reform brought before the Association. It was moved that Dr. WELLFORD'S suggestions touching the licensing power, should be referred to a committee, who should bring the matter fairly before the profession, and, if necessary, memorialize the legislatures of the several States to carry out the wishes of the Association. Immediately there was a professorial onslaught, and the subject was laid upon the table.

It was then moved that no delegates be received in the Association from medical schools, which give two courses of lectures annually, each of which counts towards a degree. Again there was a flutter among the ranks of the lower orders of professors, who teach (!) all the year round, and, on motion of Dr. ATLEE, of Pennsylvania, the resolution was laid upon the table.

In this connection, Dr. SAMUEL JACKSON mentioned an instance of a mechanic of Phœnixville, Pa., who had unsuccessfully attempted the vocations of cabinet-maker and grog-shop keeper, and had subsequently practiced, with popularity, as a Thompsonian quack. It was suggested to him that it was desirable that he should possess a diploma. He left his residence, and returned in one week, with the diploma of a Philadelphia Medical School. In reply to interrogations from various delegates, the eminent professor stated the name of his informant—a respectable practitioner of Phœnixville, and the name of the institution by which this diploma was granted,—the Philadelphia College of Medicine, commonly known as "McClintock's School." Dr. BRYAN announced himself as the representative of this institution, but no motion was made to deprive him of his seat.

Various efforts were then made to induce the Association to recommend Medical Schools to require their graduates to subscribe to a pledge to submit to the revocation of their diplomas upon conviction of having knowingly violated the Code of Ethics of the National Association. There was such an entire absence of concert among the friends of reform, that these efforts were easily frustrated.

The subject of amendments of the Constitution now came up, and the delegates from Virginia presented the resolutions of the Society of that State, urging such changes in the Constitution, as should exclude from representation in the Association, all colleges and schools which do not conform to its recommendations on the subject of medical education. Dr. STEVENS, of New York, moved the indefinite postponement of all amendments, and it was decided that this motion was not debateable. Accordingly, it was carried by a triumphant majority, and the schools are left to the tranquil pursuit of their glorious objects for another year.

In thus reviewing the late session of the American Medical Association, and arriving at the painful conviction that it accomplished nothing of importance or value on that occasion; that, on the contrary, its deliberations have had an injurious effect, in showing its impotence to effect those radical reforms expected of it, we desire to impress upon our readers the necessity for active and individual exertion in behalf of the interests of the profession. The voice of public opinion will be far more effectual in suppressing the evils which beset us, than the unheeded remonstrances of associations.

We know that it is far easier to tear down than to build up, to point out defects than to correct them, and our expectations of improvement are not very sanguine. Still, we believe it is in the power of individual physicians, by properly cultivating the noblest and most useful of human pursuits, by jealously guarding against every temptation which can lower the dignity of their calling, by refusing to receive students whose character, or want of preliminary education, unfit them for scientific studies, to elevate their profession in their own eyes, and in the opinion of the world."

